# Walls-in-one: usage and temporal patterns in a social media aggregator

**DOI:** 10.1007/s41109-016-0009-9

**Published:** 2016-07-11

**Authors:** Matteo Zignani, Azadeh Esfandyari, Sabrina Gaito, Gian Paolo Rossi

**Affiliations:** Universitá degli Studi di Milano, Dipartimento di Informatica, Via Comelico 39/41, Milan, Italy

**Keywords:** Multiplex network, Social media aggregators, Degree correlated networks, Burstiness, Username usage

## Abstract

The continual launches of new online social media that meet the most varied people’s needs are resulting in a simultaneous adoption of different social platforms. As a consequence people are pushed to handle their identity across multiple platforms. However, due the to specialization of the services, people’s identity and behavior are often partial, incomplete and scattered in different “places”. To overcome this identity fragmentation and to give an all-around picture of people’s online behavior, in this paper we perform a multidimensional analysis of users across multiple social media sites. Our study relies on a new rich dataset collecting information about how and when users post their favorite contents, about their centrality on different social media and about the choice of their username. Specifically we gathered the posting activities and social sites usage from Alternion, a social media aggregator.

The analysis of social media usage shows that Alternion data reflect the novel trend of today’s users of branching out into different social platforms. However the novelty is the multidimensional and longitudinal nature of the dataset. Having at our disposal users’ degree in five different social networks, we performed a rank correlation analysis on users’ degree centrality and we find that the degrees of a given user are scarcely correlated. This is suggesting that the individuals’ importance changes from medium to medium. The longitudinal nature of the dataset has been exploited to investigate the posting activity. We find a slightly positive correlation on how often users publish on different social media and we confirm the burstiness of the posting activities extending it to multidimensional time-series. Finally we show that users tend to use similar usernames to keep their identifiability across social sites.

## Introduction

In the last decade large datasets describing online social networks (OSNs) have been made available together with an extensive literature which enabled a comprehensive description of OSNs. Most of these studies assume that people connect and establish relationships on a single social platform. This approach, however, enables the achievement of a partial representation of the online social behavior of individuals Ruths et al. (2014). It is becoming evident that individuals are used to express their sociality through multiple layers ranging from face-to-face and on-phone communications to a variety of online social networks. Especially in their online life, users have access to a wide portfolio of social platforms which allow them to differentiate their interests and convey diverse contents. As a result, a user can be registered to many social media and interact with her/his friends’ circles throughout different channels. For instance, a user can share her/his photos on Instagram, organize an event on Facebook and maintain the relationships with workmates on LinkedIn. These arguments are quantitatively reported in a survey conducted in September 2014 ^1^ where it has been highlighted that the adoption of multiple social platforms is on the rise. In fact, 52 % of online users now use at least two social media, representing a significant increase from 2013, when the figures were close to 42 %. The diversification in the usage of the social platforms, in conjunction with their specialization, is causing a fragmentation of users’ identities among diverse social media. This process of identity fragmentation makes the understanding of the online behaviors more difficult since data are split and need to be matched and fused.

In this paper we move towards the reduction of the above segmentation by facing with the new challenge of giving a complete and compelling picture of people’s online behavior. To this aim we move within the multidimensional or multiplex network theory Magnani and Rossi (2011); Bródka et al. (2012); Berlingerio et al. (2012); Kivelä et al. (2014); D’AGostino and Scala (2014) since it provides the tools and the models which better capture and measure the interplay/correlation among the social sites users adopt. Specifically the scenario we are dealing with is well represented by a multigraph Zignani et al. (2015), a special case of an heterogeneous information network Sun and Han (2012). So far multiplex network theory has been applied to different real case studies; from citation Li et al. (2016a), co-author Sun et al. (2011) and conference-author networks Sun et al. (2009) to power grids Brummitt et al. (2012), economic Li et al. (2016b); Li et al. (2014); Barigozzi et al. (2011) and biological networks Bauch and Galvani (2013). However most of these studies assume the underlying multiplex network is static or well-known dynamical processes occur onto them. On the contrary, little is known about the temporal interactions of people when they have different communication media available, especially in the online world. So the main goals of this paper are *i*) applying the multiplex network theory to understand the on-going phenomenon of the adoption of multiple social site; and *ii*) measuring how this process impacts on the interaction dynamics.

In practice, the study of the behaviors of people across different online social networks is at its very beginning. In fact, while many works have been published about profile matching algorithms across sites Zafarani and Liu (2013); Carmagnola and Cena (2009); Vosecky et al. (2009); Goga et al. (2015), only a few works Benevenuto et al. (2009); Meo et al. (2013); Abel et al. (2013); Magnani and Rossi (2013) analyze user behavior across sites, but they focus on specific behaviors as tagging or changes in clickstream.

One of the main hindrance in this field is the lack of datasets enabling the analysis of the single individual’s behavior across different sites. This criticality can be ascribed to a variety of motivations. First of all, people are unwilling to make all information about their online social life public and, secondly, social site providers have less of an interest in providing tools to integrate other social platforms. To overcome the data problem, in this paper we rely on the services offered by social media aggregators. A social media aggregator is a Web service which allows users to collect and manage different social site accounts through a single application. Most of these services offer to their users a private space, where they can share a content simultaneously on multiple media. Among these services, we retrieve data about users and their social sites from Alternion^2^, because allows users to make information about their social sites public, unlike most of social media aggregators. Thus, it is possible to collect data about their profiles with personal public information and the posted contents.

This paper offers a first data-driven contribution to the study of people behaviors across social sites while addressing the following research issues:

- **Q1**: How common is the use of multiple social media sites? Does the same hold for active users?
- **Q2**: Is a person’s popularity uniform, i.e. more or less equally distributed across the social sites in use?
- **Q3**: Is users’ production alike in different social media ? Do most active users behave similarly on different media?
- **Q4**: How do people manage their posting activities during the day? Do they post on multiple social media every day or do they alternate in the choice of the publishing platforms?
- **Q5**: Are users coherent in the choice of their usernames, keeping their identifiability across social sites?

By answering the above questions the paper introduces the following main findings and contributions. On the one hand, some findings confirm that the adoption and the active usage of multiple social sites is gaining momentum and maybe be studied through social media aggregators. On the other, we find that a full multiplexity of the interactions and posting activity is difficult to reach in short periods (day) but more evident in longer.

- **Membership distribution across social media.** The way users distribute their membership across sites has been recently studied in Zafarani and Liu (2014) and has been the subject of a few market surveys. In the third section we confirm and extend these results by means of a more recent dataset, which consequently relies on the today’s most popular social websites. The usage of multiple social platforms, raising in 2008, is now strengthening and social media aggregators offer a chance to collect data about this phenomenon.
- **Popularity across social media.** By performing a correlation analysis we investigate the maintenance of users’ popularity across social sites. The analysis led to not straightforward results and indicates that user’s popularity in a given social site barely corresponds or does not correspond at all to his/her popularity on another social platform. The lack of a strong degree correlation has been observed in different contexts such as online/offline interactions Gaito et al. (2012a), virtual worlds Szell et al. (2010) and other multiplex networks Nicosia and Latora (2015). Here we observe the same phenomenon in online social platforms.
- **Posting activity across social media.** We aim at understanding whether an active and productive user on a social media preserves his/her aptitude on the other social media. Correlations between the posting rates on different social sites show that an easy answer is not possible, although we measure slightly positive correlations for many couples of social platforms. These results represent one of the main novel insights of this paper since little is known about users’ engagement on multiple social media.

In general the last two findings, presented in the fourth section, stress the fact that people, in a multiplex scenario, change their importance and preferences from medium to medium. The ability of handling some particular media better than others, the different interests and the usability of the services may be the reasons behind this behavior.

- **Burstiness in temporal patterns.** We have analyzed the posting inter-event times both aggregated on all social sites, to get an overall picture of the activity dynamics of users, and a per site evaluation, to get temporal patterns specific to a social website. This way, we evaluate the dynamics of the online activity by a true multidimensional approach. We discovered that the posting activity on online social media is bursty and highly heterogeneous. In particular the bursty behavior of the aggregated sequence is the union of bursty posting event sequences on different social media. Moreover a period of high activity in the aggregated sequence does not imply that the user is highly productive in each social media along the same period.
- **Temporal multiplexity.** We introduce the *post multiplexity* index to measure the propensity of a user of being multidimensional. Results show that most of the users tend to prefer a single media per day while sometimes they adopt multiple social media for posting.

The above results, presented in the fifth section, represent the first steps towards a multidimensional approach in the study of human dynamics Szell and Thurner (2010) in communication and social networks. Some attempts to extend and model bursty dynamics can be found in Quadri et al. (2014) and Jo et al. (2013), however the layers are few or do not really represent communication channels. In this paper, thanks to a large set of social media, we highlight the multidimensional bursty nature of human dynamics in multiple social sites. Specifically, we show that the overall burstiness is a consequence of a complex mixture of non-stationary interests to the chosen social media.

- **Patterns in username usage.** We evaluate how users are coherent in the choice of their username across social sites. Results show that users often maintain the same username across different social websites, but are more likely to change them among websites whose norms and scopes are different.
- **Alternion datasets.** The above results rely on two new collected datasets which capture multidimensional and longitudinal information about how online users behave across multiple social platforms. To the best of our knowledge, they represent the most updated available datasets which combine posts, their contents and the multiple profiles of a large set of people. The Alternion dataset allows us to quantify the multiple platforms usage without requiring a periodical survey; on the other, additional information about posts could be extracted to verify whether the same observations hold for users’ engagement. Furthermore, posts provide the temporal information necessary to the study of multidimensional human dynamics in online social networks.

The aforementioned results support the increasing awareness that single social site studies provide a very partial description of human social behavior which effectively needs a multisite approach to be described and fully understood. To this aim, social media aggregators, such as Alternion or About.me, represent data collection to be deeply analyzed, provided that we take into account the bias given by the typical users of these platforms.

## Methodology

Nowadays people have at their disposal a wide selection of online social sites, each having its own peculiarity. This way, the adoption of multiple social platforms by the same person is becoming increasingly spread. Now people can exploit and combine their favorite social media according to their needs. For example, a tourist could share his/her position by Foursquare, meanwhile s/he uses Twitter to communicate his/her mood and shares on Instagram a selfie with the “Gioconda”.

At the same time, people have recently shown a growing interest in tools managing their online life in a centralized way, i.e. social media aggregators. Social media aggregators allow users to combine their own identities into a single profile by gathering their online activities from different social platforms, such as Twitter, YouTube, LinkedIn, Facebook, and many others. The aggregation is enabled by APIs provided by social networks, so that people may have control on the data the aggregation platform can access.

Most of these services offer a private space to the users, where they can share a content simultaneously on multiple media, but profiles with public contents and public API are not available. The media aggregator Alternion ^3^ stands out because, unlike most of similar services, allows users to decide which information about their social sites - including profile information and contents - can be made public. Furthermore, the service retrieves data from more than 200 social sites and manages social relationships among Alternion profiles.

### Data collection methodology

**Fig. 1:**
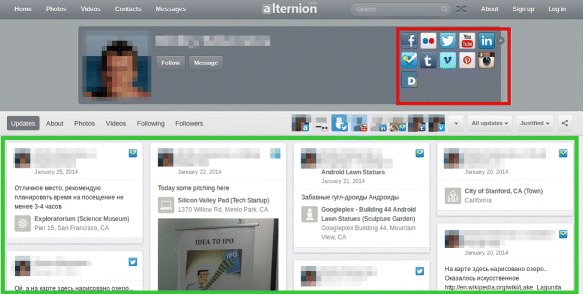
Example of a profile page in Alternion. The *red box* highlights the social platforms shared by the user. A pop-menu shows the number of relationships in each social network. The green box highlights the area containing all the public posts gathered by the API of the services

Since the service does not expose a public API, we developed a crawler to retrieve the Alternion profiles and their public updates. An example of an Alternion profile page is shown in Fig. 1. The page exposes the social sites associated to the Alternion identity. Each icon links to the relative profile and shows the username chosen in the target social site. The "Updates" tab reports the public posts grouped by social sites. The content of each post highly depends on the information released by APIs.

We collected registered users by exploiting a search function which returns a different set of 40 random users every two minutes. Data collection started on October 2014 and resulted in 19.680 distinct profiles from different countries and in different languages. From a user profile we extract the set of social sites a user associates to the profile and all the public contents s/he had published since the registration date. For each content we gather information about the social media used to post it, the content itself according to the format returned by the APIs and the publication date. Furthermore, not to overload the service and to respect politeness, we limit the number of contents for each user to 10.000 at most. In practice, the dataset contains, for each individual, a list of her/is favorite social sites and a multidimensional time series of posts, where each dimension corresponds to a specific social site. All the analysis, but username patterns reported in the last section, will be performed on this dataset *D*1. In addition, each profile reports the degree of the user on the social networks that make this information available. However, we are not able to get the node neighborhoods since Alternion does not return the neighbors’ usernames. Consequently, we are not able to build any network from the information provided by the social media aggregator. Since we may be able to build different types of relation by exploiting implicit links such as mention, we model our dataset by a directed multigraph $\mathcal {D}=(V,E,D)$. Each user (identity) is an element of *V*, while *E*⊆*V*×*V*×*D*, with *D* the set of social media in Alternion, is the set of directed multi-edges. Finally, we associate to each user *u*∈*V* a sequence of timestamped events (*t*,*p* *e*_*d*_), where *pe* represents a post published on the social media *d*∈*D*.

For the study of username patterns only, we collected a different dataset, *D*2, whose characteristics are specifically designed to this aim. We adopted a more targeted sampling approach by collecting 15.000 profiles with an English first name ^4^ to make the text analysis of usernames more affordable. This way we maintain the alphabet accordance between the username (typically ASCII character) and the information about the identity (first and last name are usually written in mother tongue). Finally, the dataset *D*2 contains for each individual a list of her/is usernames.

### Dataset characteristics and representativity

**Fig. 2:**
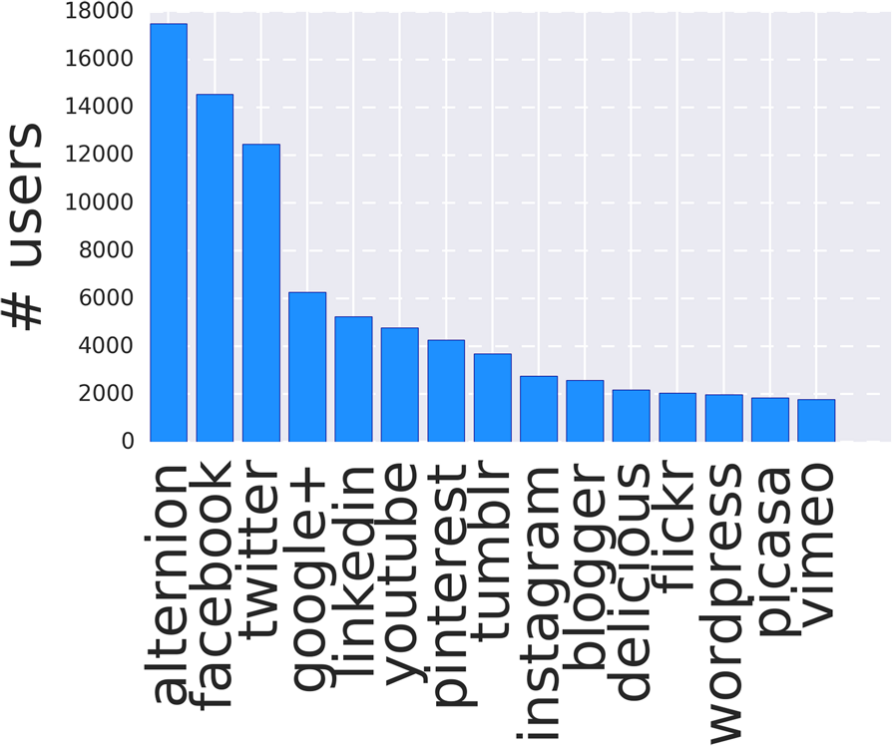
Social site adoption. The number of users for each social platform

**Fig. 3:**
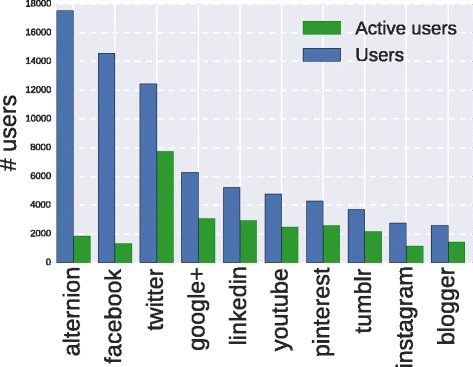
Social site engagement. The number of users/ active users for the top ten social sites. Active users represent people who have published at least one post on a social site

The dataset *D*1^5^ contains the profile information of 19680 users, each reporting the platform Alternion among her/is social sites. Specifically the number of social platforms amounts to 152, from the most famous Facebook, Twitter to the less common Discus or Zazzle. In Fig. 2 we report the number of users who share their profile information of a social site, while in Fig. 3 we report the number of users for the top ten social platforms. As expected, and ignoring Alternion, Facebook is the most used social site and the top 10 correspond to the most popular social media.

In Fig. 3 we also report the number of active users per social site. Active user denotes a person who has published at least one content on the profile page. About half of the profiles (9829) are active and do prefer Twitter as publishing media. Although Twitter result is expected due to the Twitter intrinsic nature (interest and media broadcasting network), LinkedIn and Google+ are more favorite than Facebook when we consider active users, even if the continuous releases of new versions of the Facebook API may influence the available information. Anywise we generally note that the users who actively adopt social platforms always represent a fraction of the registered users.

**Fig. 4:**
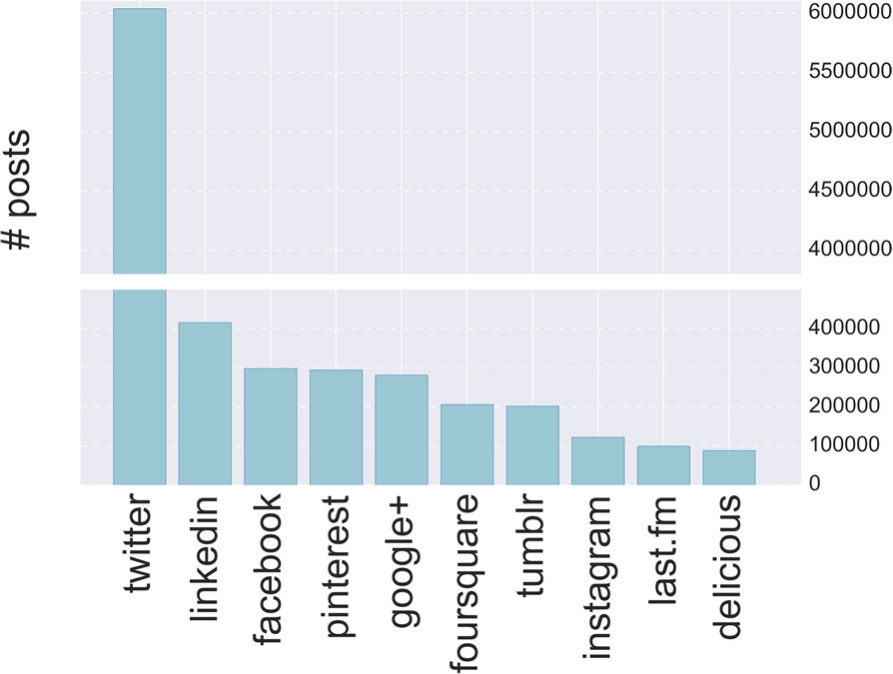
Posting activity. The number of posts for each social site

We obtain a deeper understanding if we combine the above results with those presented in Fig. 4, where we report the number of posts grouped by social sites. Here we note that Facebook active users are more productive than Google+ users. In fact, although they are fewer, Facebook users publish more or less the same amount of posts as Google+’s. Finally, results in Fig. 4 confirm the predominant role of Twitter in the production of contents and posts, i.e. more than 6 million posts over an overall amount of more than 8.5 million posting events.

**Fig. 5:**
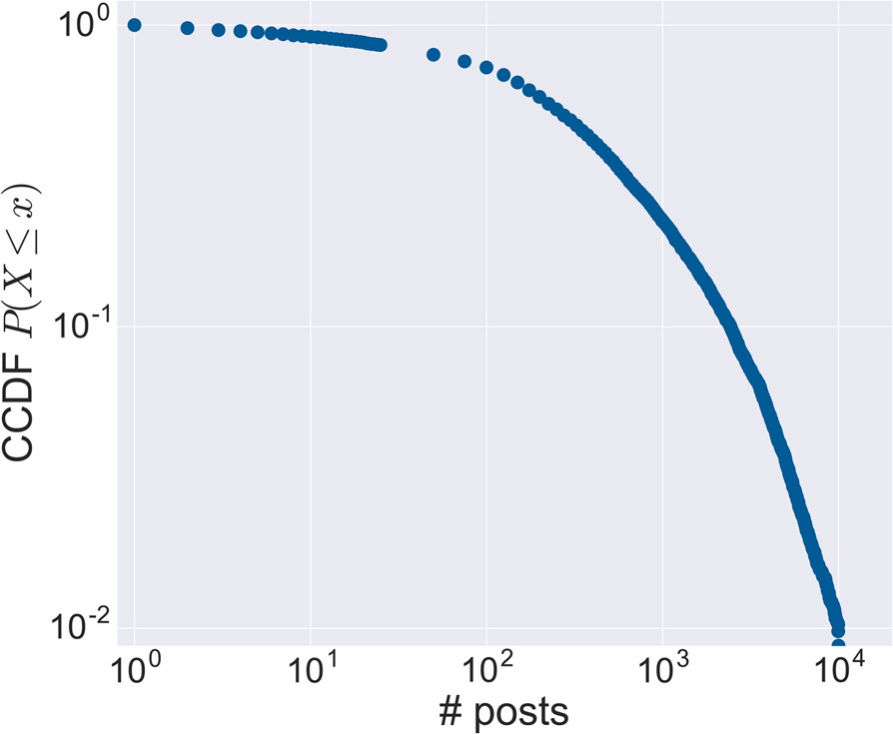
Posting activity. The complementary cumulative distribution function (CCDF) of the number of posts published by each user

As regards the posting activities of the active users, in Fig. 5 we show the distribution of the number of published contents per user. The post sampling covers the time interval from 10 July 2005 to 10 November 2014. The distribution seems to obey to a heavy tail with exponential cut-off. During the examined period, we observe that users have been quite productive: on average, users have published about 800 different contents and half of the users have produced more than 300 updates.

The above results highlight that Alternion effectively captures a representative sample of today’s users of social media sites, in terms of favorite social sites and publishing media as compared with public available data^1^ on social media usage. Indeed, the usage proportion across the social sites are almost equal to those collected by aggregating the official statistics released by the different social platforms. Despite data show an alignment between the Alternion users and the users of the different social media, the representativeness issue of the dataset still persists. For instance, users’ demography is unbalanced towards western and English-speaking countries, the contents posted on the Alternion profile depend on the API provided by the social media. Lastly, the adoption of social media aggregators is more common and useful to an audience active on social media. Nevertheless, it represents the most recent dataset able to capture how users behave across multiple sites and an easy tool for studying the on-going phenomenon of the multiple adoption of social platforms.

## Usage of multiple social sites

In a survey conducted in September 2014^1^ it has been highlighted that the adoption of multiple social platforms is on the rise. In fact, 52 % of online users now use at least two social sites representing a significant increase from 2013, when the figures were close to 42 %. On the one hand the Alternion dataset allows us to quantify this phenomenon without requiring a periodical survey; on the other, additional information about posts could be extracted to verify whether the same observations hold for users’ engagement. Indeed, being registered to a service does not always imply using it.

**Fig. 6:**
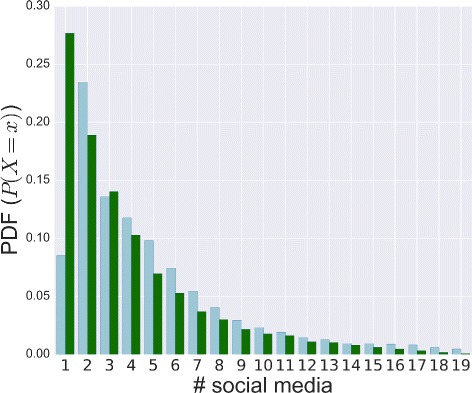
Social sites per user. The PDF of the number of social sites joined by each user (*blue*)/active user (*green*)

To answer Q1 - *How common is the use of multiple social sites? Does the same hold for active users?* - we analyze how users are distributed across sites. The main goal of a social media aggregator is to group different social sites into a single access point. This characteristic allows us to compute how many different social websites people are able to manage. How users distribute their membership across sites has been studied in Zafarani and Liu (2014) only, where authors showed that it follows a power-law distribution from a dataset gathered in 2008. Our goal is to extend that result, by confirming it on a more recent and larger dataset, which relies on the current social platforms. In Fig. 6 we report the probability distribution function (PDF) of the number of sites joined by a given person. On average a user is simultaneously registered on 5 social sites, while more than 95 % of users have joined 17 platforms at most. Alternion results are consistent with the aforementioned survey; indeed about 56 % of Alternion users use at least three different social platforms^6^.

The behavior of active users gives a more direct and clear evidence of the adoption of multiple sites. In fact the publication of contents on a specific social media confirms its adoption in practice and it is strictly related to the user’s engagement. This way, we compute the distribution of the number of social platforms being simultaneously used by each active user. As shown in Fig. 6, the observations about the multiple usage get stronger: 73 % of active users publish on at least two social networks. In general, the propensity to adopt multiple social sites is becoming increasingly ingrained amongst online users, especially if we consider the effective publishing on different platforms.

**Fig. 7:**
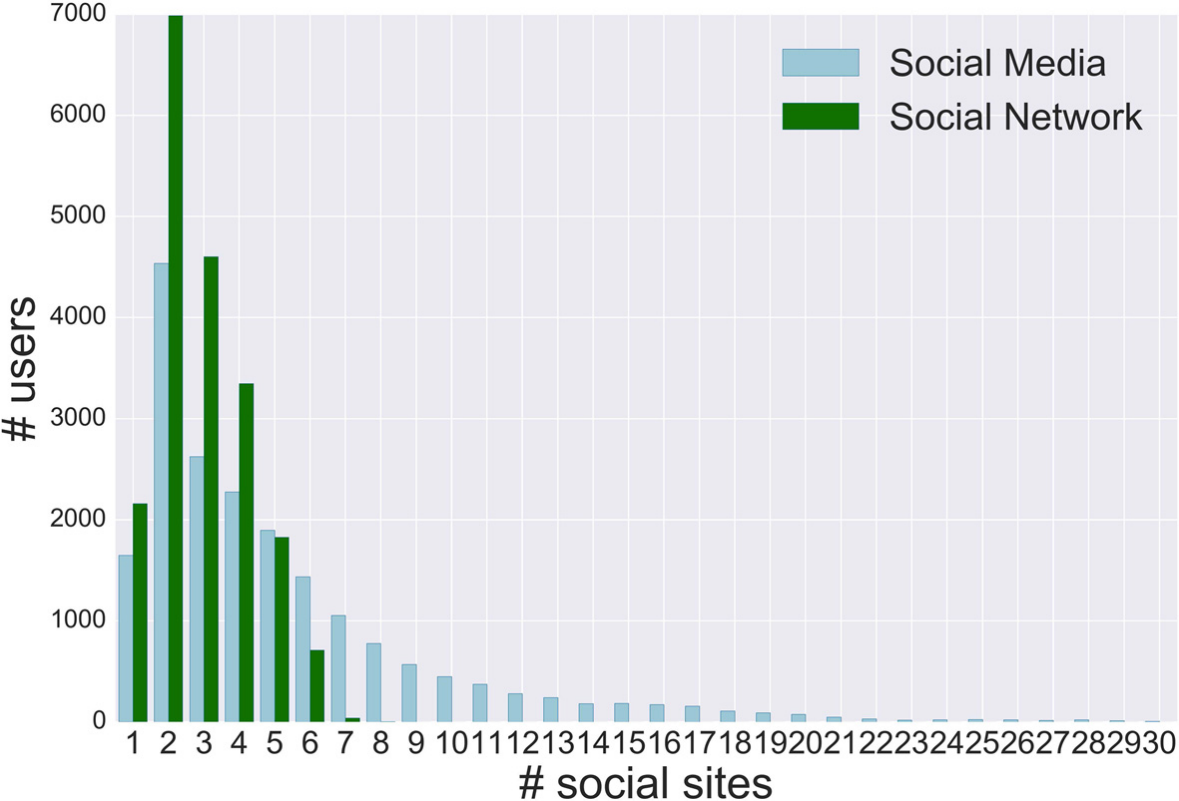
Posts and number of sites. The average $\bar {p}$ as a function of the number of sites users have joined. The error bars report the standard deviation

The above analysis on the people’s propensity to be active on multiple social media does not take into account the amount of contents published on each platform. To this aim, we analyze the productivity of the users who join more the one site simultaneously. Let *p*_1_,*p*_2_,…,*p*_*n*_ indicate the number of posts of a user on the *n* sites where s/he is active and $\bar {p}=\sum p_{i}/n$ the average number of posts per site. For each active user we compute $\bar {p}$ and group them by the number of multiple sites they have joined; then we calculate the mean and the standard deviation of $\bar {p}$ for each group. In Fig. 7 we report the above quantities as a function of the number of sites. The figure indicates that *i*) the more sites a user joins, less posts for site on average s/he publishes, and *ii*) users who post on few sites are more heterogeneous in their activity since the standard deviation decreases as the number of sites increases. We suppose that the first remark is a consequence of the limited human resources in terms of usable time and ability of handling multiple tasks at the same time Miritello et al. (2013).

**Fig. 8:**
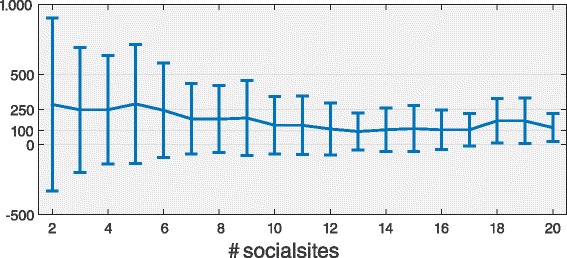
Social media and networks. The PDF of the number of social sites joined by each user subdivided in social media and social networks

Finally, in Fig. 8, we show the number of users who have joined different social sites by considering two classes: social media and social networks. The former denotes social sites where relationships are implicit, the latter indicates social sites where relationships are explicitly defined. These figures show that more than 90 % of users have registered to at most 5 sites and some of them share information on more than 10 social media. In general we observe that it is more likely to adopt social media than social networks.

## Centrality and activity correlation across social sites

To answer Q2 - *Is a person’s centrality uniform, i.e. more or less equally distributed across the social sites in use?* - we treat the user degree on different social sites as an index of centrality in the network. This information, reported on the Alternion profile, is not available for every site since just a few APIs allow to retrieve this information. So, we limit our analysis on the degree to Facebook, Twitter (in/out), LinkedIn and YouTube (in/out).

**Fig. 9:**
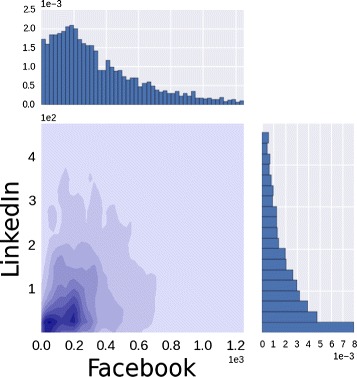
Facebook and LinkedIn. The joint probability distribution estimated by kernel density method

Q2 corresponds to verify if statistically significant correlations between the degrees of the same group of users in different sites exist. The presence of these correlations measures whether or not popular users in one site maintain their centrality across media. If we denote ${k_{u}^{s}}$ as the degree of the node *u* in the site *s*, we can evaluate the degree of correlation between pairwise social sites by adopting different methods Boccaletti et al. (2014). First we compute, for each pair of social sites, the joint distribution *P*(*k* ^*s*1^,*k* ^*s*2^) to obtain a characterization of the relations between the degree sequences. For example, in Fig. 9, where we show the joint distribution for Facebook and LinkedIn, we observe a slightly positive correlation, especially in the left bottom part of the distribution (dark blue regions). In particular people with about 200 friends in Facebook and about 20/25 relationships in LinkedIn are more likely than others.

**Fig. 10:**
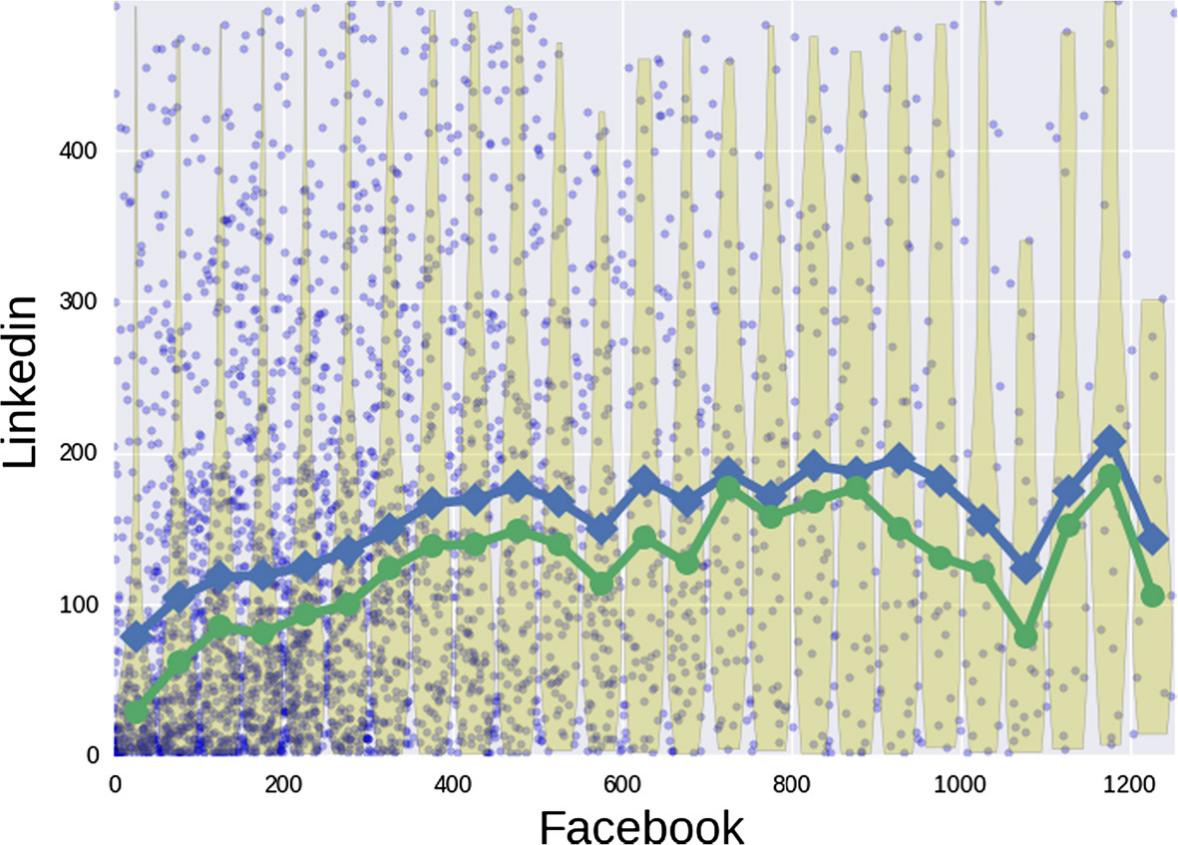
Facebook and LinkedIn. The conditional distributions of the LinkedIn degree given Facebook degree. For each 50 size bin, we show the degree distribution through the violin plot with its average (*blue*) and its median (*green*)

**Fig. 11:**
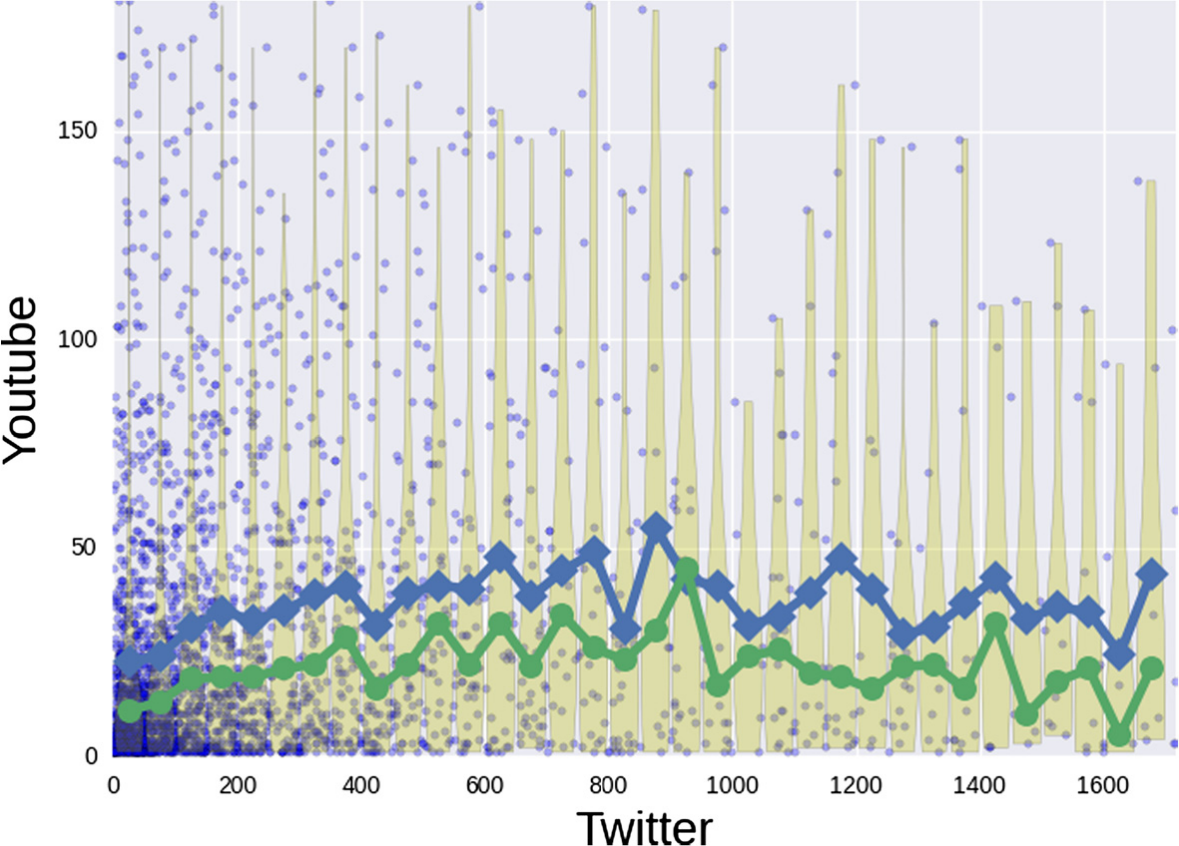
Twitter and YouTube. The conditional distribution of the out-degree in YouTube given the Twitter out-degree. For each 50 size bin, we show the degree distribution through the violin plot with its average (*blue*) and its median (*green*)

By analyzing the joint distributions only, it is difficult to compare the relations between a social site against the other ones. To this aim, we compute the average degree of a node in a social site *s*_1_ conditioned on the degree in the site *s*_2_. The resulting computation for LinkedIn conditioned on Facebook has been shown in Fig. 10. In this case we observe an initial increase of the average degree in LinkedIn as a function of the Facebook degree up to about 500 friends. Then we observe a more unstable trend also due to a low number of data points. In the Fig. 11 we report a more pronounced lack of correlation involving the out-degree in Twitter and YouTube. Here we cannot establish an increasing relation between the out-degrees, so people how follow many users in Twitter would not follow the same amount of channels in YouTube. The specificity of the two social platforms, i.e. news broadcasting and video sharing, could be the cause of the weak correlation.

**Fig. 12:**
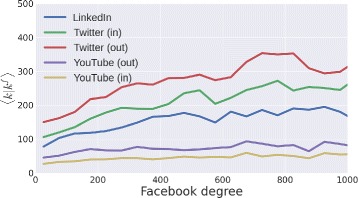
Degree dependencies. The average degree in LinkedIn, Twitter and YouTube as a function of the degree in Facebook

Keeping Facebook as conditioning social site, we observe that the average degree increases in some media like LinkedIn and Twitter, while the in/out degrees in YouTube are uncorrelated with the Facebook degree; as shown in Fig. 12. The comparison between the degree and the in/out degree is dictated by the inability to extract mutual links (more similar to a friendship link in Facebook or LinkedIn) since we can only retrieve the counting of the followers/followees in Twitter and YouTube.

**Fig. 13:**
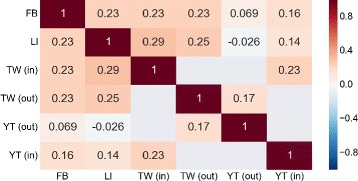
Degree rank correlation. The correlation matrix among the degree sequences in the different social sites

Finally, to get an overall picture of the pairwise degree correlations, we apply a rank correlation analysis on the different pairwise sequences. Rank correlation analysis allows us to test if the ranking induced by the different degrees is similar or not. As a rank correlation method, we compute the Kendall’s rank correlation coefficient *τ*_*b*_^7^ on the ranking induced by the degrees. In Fig. 13 we visualize the rank correlation matrix, where each row (column) corresponds to a different social site. A strong positive correlation does not exist, rather the scenario is multifaceted. In most pairs, there is only a limited positive correlation (0.1−0.23) between degree centralities. This means that users may have a very different centrality across the services, i.e. a single user might be an hub on one system and loose part of its hubbiness on the other. One reason may rely on the different goals of the services; whereas LinkedIn is business-oriented or Twitter is an interest network, Facebook incorporates all the previous features. So, for instance, LinkedIn may only capture a part of the Facebook friends.

The above conjecture cannot be verified since we are not able to compute the intersection between a node’s neighborhoods in different social networks (information not provided by Alternion). However we can quantify and analyze the difference between the neighborhoods in terms of their size. To this aim, given a user *u* and two sites *s*_1_ and *s*_2_, we define the *friend deviation* $\Delta k^{s1s2}_{u} $ as:

1 $$ \Delta k^{s1s2}_{u}=k^{s1}_{u}- k^{s2}_{u}  $$

**Fig. 14:**
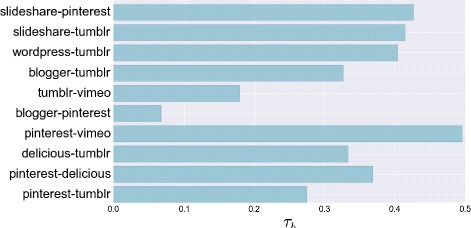
Friend deviation. The friend deviation scores sorted by decreasing order

We compute the friend deviation between Facebook and Linkedin($\Delta k^{FL}_{u}$), Facebook and Twitter($\Delta k^{FT}_{u}$), Facebook and YouTube($ \Delta k^{FY}_{u}$), Twitter and LinkedIn($\Delta k^{TL}_{u}$) and Twitter and YouTube($\Delta k^{TY}_{u}$) for the users who have joined them. We report the trends of the friend deviations in Fig. 14 sorted in decreasing order. We observe that $\Delta k^{FT}_{u}$, $\Delta k^{FL}_{u}$, $\Delta k^{FY}_{u}$, $\Delta k^{TL}_{u}$ and $\Delta k^{TY}_{u}$ are positive for 5360 users (out of 8527), 3313 users (out of 4361), 2944 users (out of 3177), 2518 users (out of 4148) and 2760 users (out of 3098), respectively. These results indicate that users in our dataset prefer to create friendships in Facebook rather than in LinkedIn, Twitter and YouTube. Moreover, users prefer Twitter rather than LinkedIn and YouTube to establish friendships. One remarkable result is that users preferring Twitter rather than Facebook have significantly more friends than those they have in Facebook.

In general maintaining the importance across social media is not a straightforward task and asks for a deeper understanding; for example it is not clear how user’s neighborhoods in different media overlap.

**Fig. 15:**
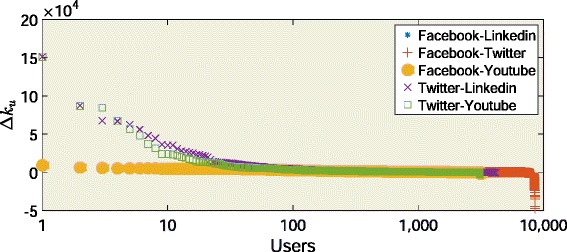
Post rank correlation. Kendall’s coefficients on the posting rate computed on some pairs of social media

Finally we apply the above methodology *i*) to investigate how often users publish contents in different social sites, and *ii*) to asses if a form of correlation exists between the amount of posts and the number of friends. For point *i*), we are verifying whether users who post a lot and often on a social platform, are equally active in other platforms (see Q3). We measure the activity level of a user within a social media by means of the posting rate, measured in number of posts per week. By considering the posting rate rather than the post count, we mitigate the effects given by the adoption of social media in different periods. We report results about the analysis of the rank correlation matrix applied to pairwise posting rate sequences. In Fig. 15 we visualize the Kendall’s coefficient *τ*_*b*_ for the most used pairs of sites. Unlike the above discussion on centrality, there is a more evident positive correlation between the posting activity across social sites. The obtained values do not mean that users are equally active on both social media, however there is a positive tendency to be active in different social sites. In general, the maintenance of the posting activity across social media is not a straightforward task, like in the degree analysis.

By the second point we wonder if users with many friends in a OSN are more active and productive than people with fewer friends. To this aim, we consider only users whose degree and posting rate are available and combine these information. The analysis of the Kendall’s coefficient *τ*_*b*_ highlights a medium positive correlation between the two variables for all the online social networks (*τ*_*b*_∈[0.27,0.4]) except YouTube (*τ*_*b*_=0.1). High degree people tend to post and publish more than those with few friends.

### A case study on 4 social media

Up to now the analysis has focused on the pairs of social media. In this section we present a particular case study that involves 524 users who have joined Google+, Pinterest, LinkedIn and Twitter. We select this subset of social media because they have the highest number of users w.r.t. other subsets of 4 elements and their users are also very active. For instance in Twitter users published 1025 elements on average, followed by LinkedIn (139.32), Google+ (137.55) and Pinterest (134.71). In fact, we observe that less than 40 *%* of users in Google+, Pinterest and LinkedIn have more than 100 posts. While in Twitter almost 87 percent of the users produced more than 100 posts.

In the light of the results about the moderate correlation among the posting rates, we wonder if, in this group, people who actively post in a service necessarily produce many posts in the other services. To this aim we denote the top 5 *%* of users in each media as most active users. Only two users are the most active in all the services. 36 *%*, 30 *%* and 42 *%* of the most active users in Twitter are also the most active in Google+, Pinterest and LinkedIn respectively. 23 *%* and 14 *%* of the most active users in Google+ are also the most active in Pinterest and LinkedIn respectively. The above observations support the presence of users who are very active pairwise but, whereas the number of sites actively used increases, the number of posts and consequently the productivity reduces as observed in the previous section.

**Fig. 16:**
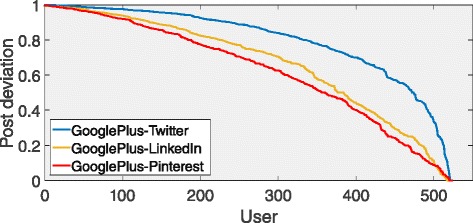
Post deviation. The deviation of the number of posts

We quantify the diversity of the posting activity across social media by defining the posting deviation $\Delta p^{s1s2}_{u}$ of a user *u* as:

2 $$ \Delta p^{s1s2}_{u}=\frac{|np^{s1}_{u}-np^{s1}_{u}|}{max(np^{s1}_{u},np^{s1}_{u})}  $$

where $np^{s1}_{u}(np^{s2}_{u})$ denotes the number of posts of user *u* on the social media *s*1 (*s*2). $\Delta p^{s1s2}_{u}$ ranges from 0 to 1: if *Δ* *p* decreases towards 0, *u* tends to publish the same number of posts both in *s*1 and *s*2. As shown in Fig. 16, where we report the posting deviation *Δ* *p* between Google+ and the other social networks, this quantity decreases linearly. From the figure emerge that users’ behavior is more variable between Google+ and Twitter than the other social networks. Only 9 *%* of users show a post deviation less than 0.5. Most of the users prefer to post on a single service. So users who laboriously publish posts in one service do not publish with the same rate in the other ones.

## Temporal patterns in the posting activity

**Fig. 17:**
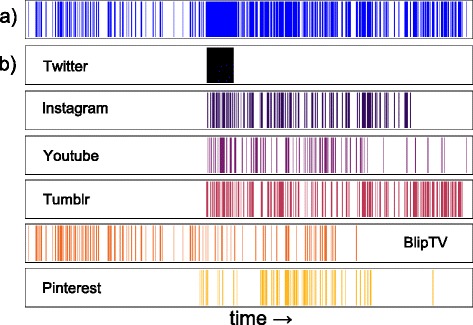
Posting activities on different media. Posting activity on different social media. In **a** the raster plot of the overall activity of a person who uses many social media simultaneously. In **b** the user’s activity split into the six social media s/he is using

**Fig. 18:**
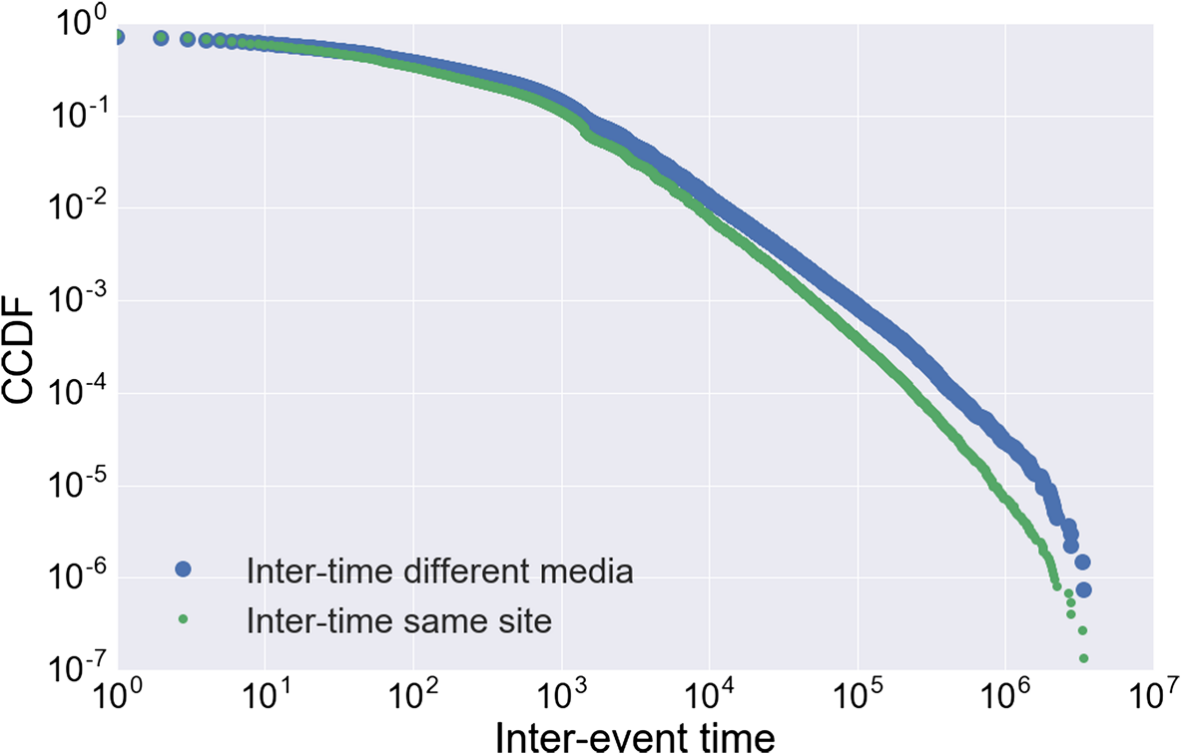
Inter-event time. The distributions of the inter-event time between two successive posts published on different media (*blue*) and between two whatever successive posts (*green*)

It is a well established result that the human dynamics on communication media are bursty and scarcely uniformly distributed in time, even removing the effect of seasonal or circadian cycles Karsai et al. (2012); Quadri et al. (2014). So we investigate whether the burstiness characterizes how people post on online social media, too. Figure 17a displays a typical posting activity of a user adopting different social media simultaneously. The overall activity presents periods where the user is almost inactive, interleaved with periods of high activity. We analyze the statistical properties of the inter-event time *Δ* *t*, i.e. the period between two consecutive posts published by the same user. Many studies have shown that the inter-event time distribution in different human processes Gaito et al. (2012b); Barabasi (2005); Karsai et al. (2012) (e.g. mobile phone call, email, direct physical contact, link formation in online social networks) is heavy-tailed. In Fig. 18 we report the distribution of the inter-event time resulting from the aggregation of the behavior of all the users. The distribution seems to obey to a heavy-tailed law and supports the hypothesis that the posting activity on online social media is bursty and highly heterogeneous.

**Fig. 19:**
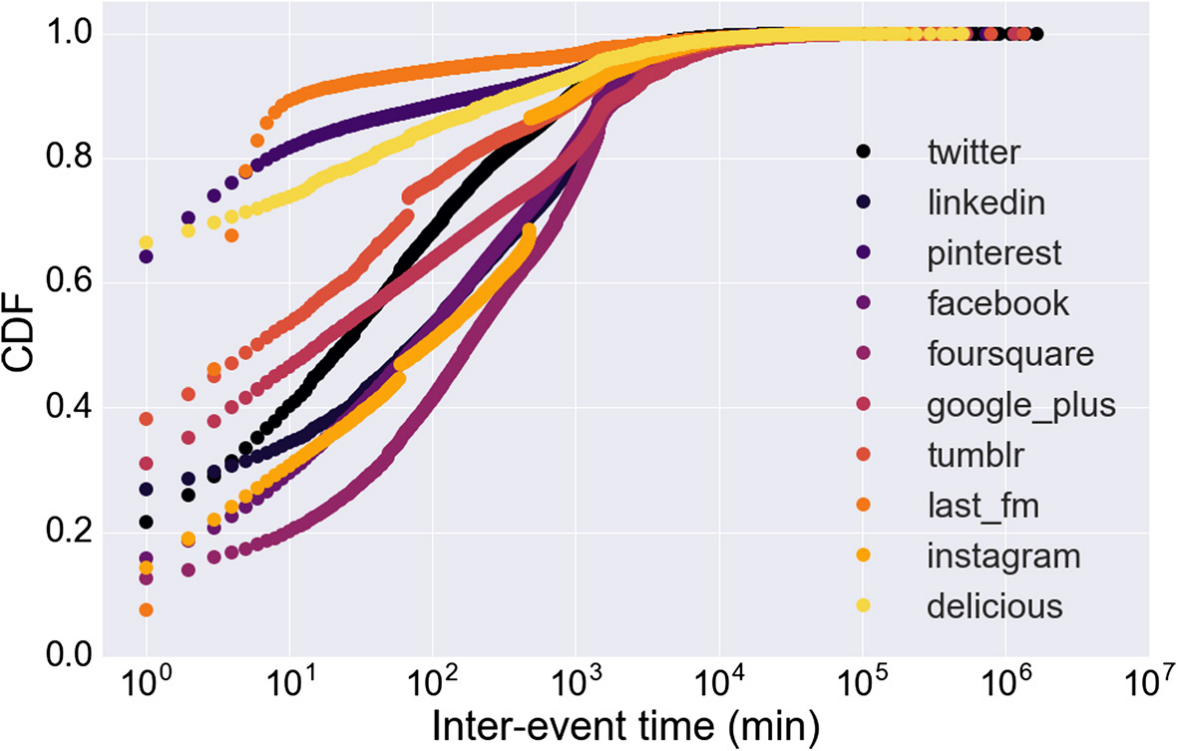
Inter-event time in the same social media. The distributions of the inter-event time between consecutive posts on the same social media. The figure reports the distributions for the most used social media. For the sake of readability we use a linear scale on the y-axis

The opportunity of tracking multiple online activities simultaneously allows us to highlight the complexity of the users’ posting activity, which appears like a compound combination of the single activities on the different social media. For instance, the sequence of events in Fig. 17 results from the union of the activity of the user in six different social media. The example emphasizes two characteristics of the event sequences: *i*) singularly each event sequence keeps an high level of burstiness, i.e. the bursty behavior of the aggregated sequence is the union of bursty event sequences and; *ii*) a period of high activity in the aggregated sequence does not imply that the user is highly productive in each social media along the same period. In the example (see Fig. 17) the user is initially active on BipTV only, then s/he begins to adopt the other social media. But the activity on Twitter ceases almost immediately and the other activities interchange like in Tumblr or Pinterest. The characteristic *i*) can be captured by the distribution of the inter-event time on each social media reported in Fig. 19. Here we extract the distributions from the social media with the highest number of posts. From the analysis of the distributions we infer two main insights: *a*) most of the distributions seem to follow a heavy-tailed law and *b*) each social media is characterized by a distribution that is far from the others. For example the probability of having a short inter-event time is higher in Pinterest, Delicious or LastFM w.r.t the other social media.

**Fig. 20:**
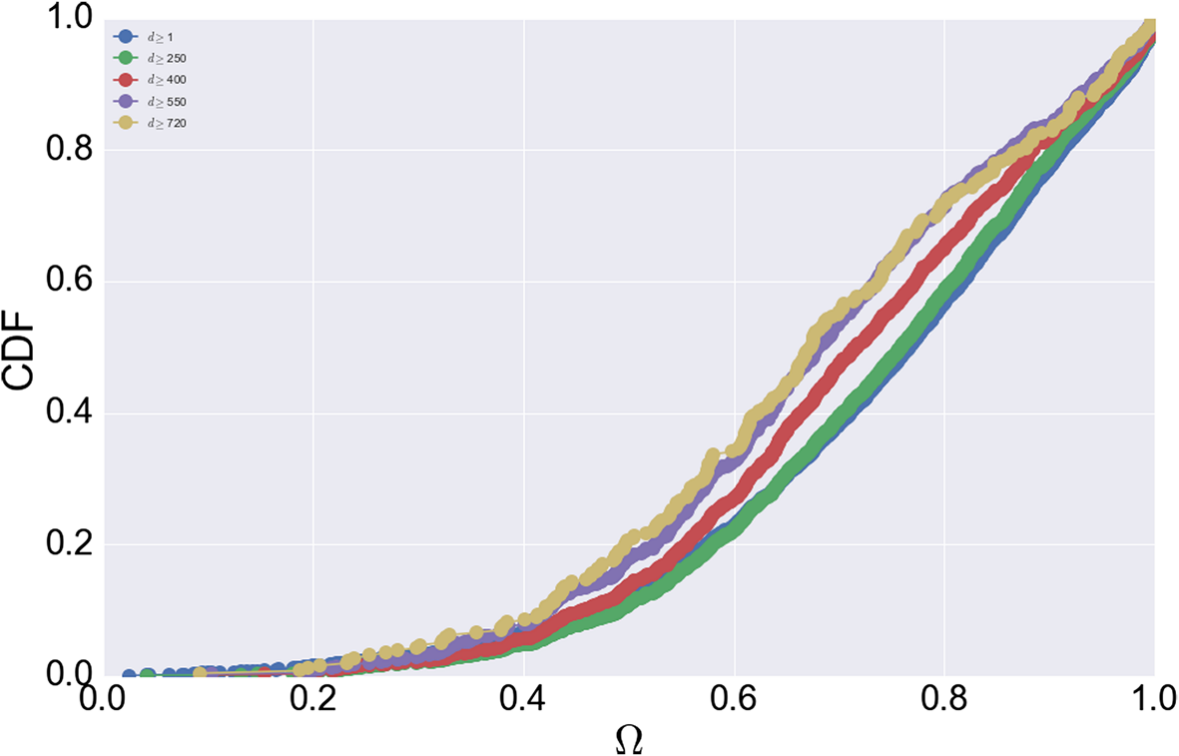
Posting multiplexity. The distribution of *Ω* for different thresholds on the number of posts per user

To verify the observation *ii*) we analyze the distribution of the inter-event time between two consecutive events that happen on different social media, a quantity strictly related to the propensity of switching among the media. The distribution shown in Fig. 19 is similar to the overall inter-event time distribution. The figure suggests that users alternate and mix their activities. The heavy-tailed distribution also indicates that the time to switch between different social media is likely to be short even if longer periods are possible. This finding suggests that during a single day a user may publish on multiple social media. To this aim we introduce an index, the *post multiplexity* *Ω*, which captures the number of days a user is active on multiple social networks. Formally, let *p*(*u*,*s* ^*i*^)=<*p*_0_,*p*_1_,…,*p*_*T*_> denote the sequence of posts published by the user *u* on the social media *s* ^*i*^, where *p*_*i*_ indicates the number of posts during the day *i*. We define a new binary sequence *p* ^′^(*u*,*s* ^*i*^), where $p^{\prime }_{i}=\delta (p_{i})$ ^8^ and the function $dm(u) = \sum _{s^{i}}p'(u,s^{i})$ which computes the number of social media used in each day. The post multiplexity corresponds to the ratio between the number of days s.t. *d* *m*(*u*)=1 and the overall number of sampling days. The index measures the propensity of a user of being multidimensional. A value close to 1 indicates than an individual uses only a social network per day. The distribution of the post multiplexity *Ω* has been reported in Fig. 20. We consider different thresholds on the number of sampling days to make the results independent of the length of the sequence and reduce the effects of less active users. The distribution suggests completely multidimensional users are quite rare as well as unmultiplex users; however most of the users tend to prefer a single media per day while sometimes they adopt multiple social media for posting.

## Patterns in username usage

**Fig. 21:**
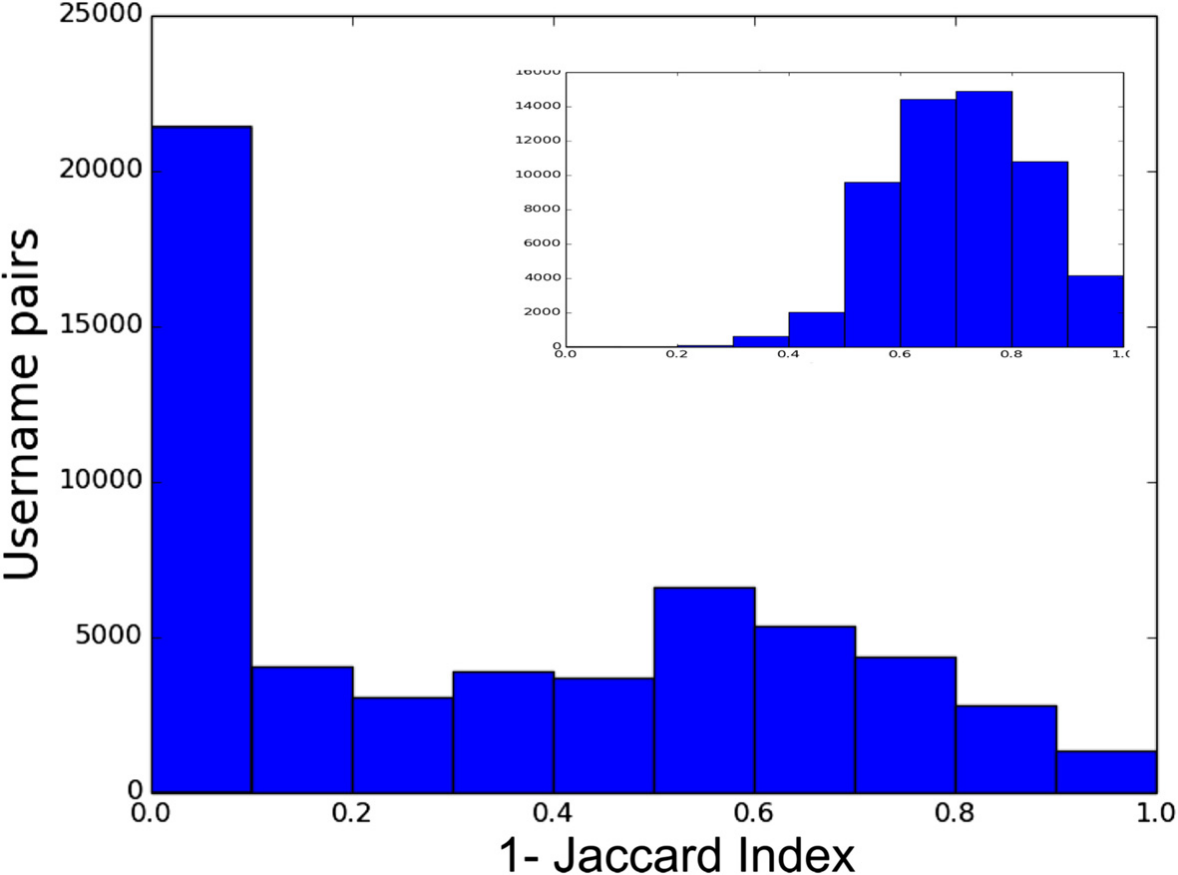
Distance distributions between usernames. The distribution of the complement of the Jaccard Index between pairs of usernames associated to the same person

**Fig. 22:**
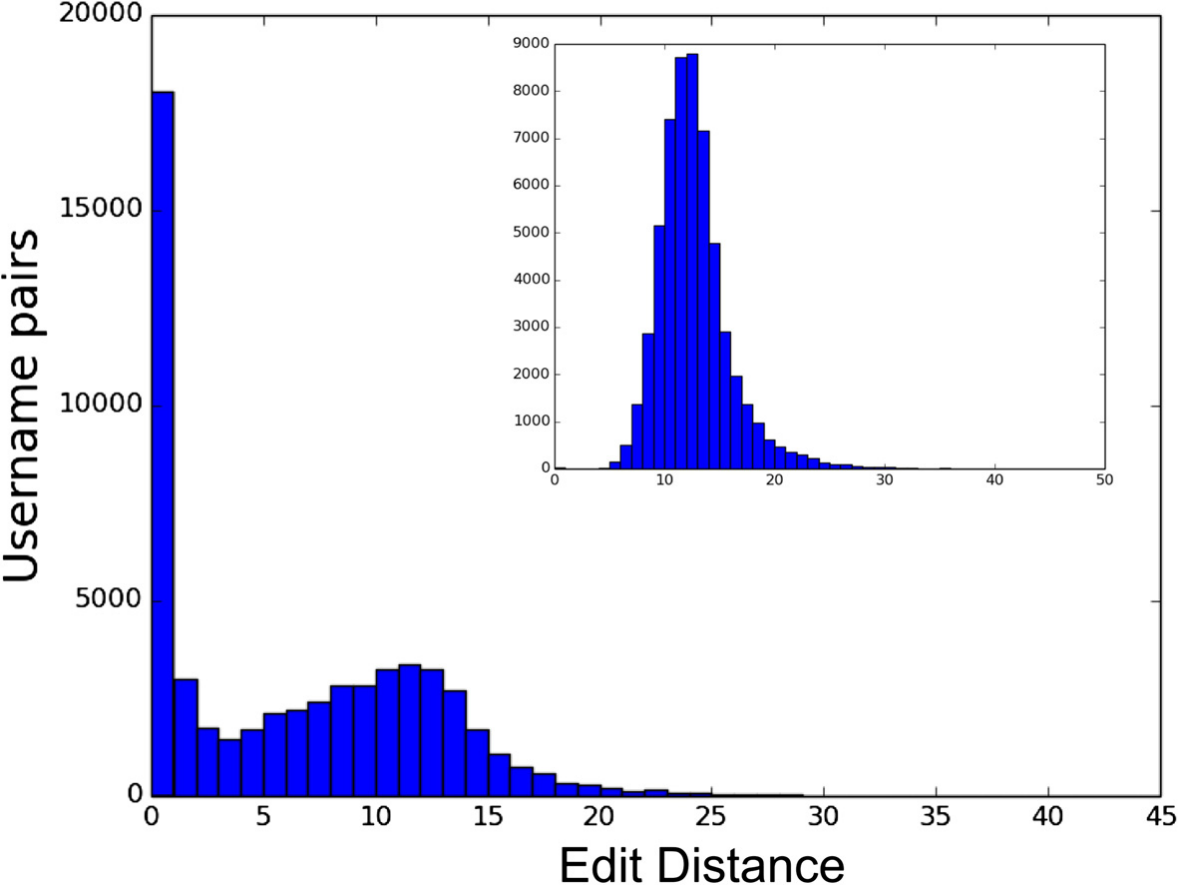
Distance distributions between usernames. The distribution of the edit distance between pairs of usernames used by the same person

**Fig. 23:**
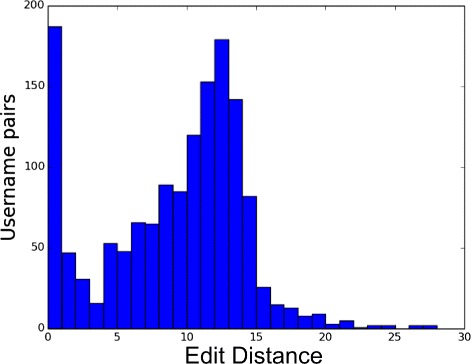
Google+ and Pinterest. The distribution of the edit distance computed on username pairs from Google+ and Pinterest

Besides providing information about people’s behavior in using and posting across social sites, the collected datasets sum up properties concerning the choice of the username in different sites. The username represents the first information provided to the social site, the first way of being identified in the site and an element in the self-presentation. In fact many behavioral aspects flow into the choice of the username, not least the limited memory capacity. On *D*2 we evaluate how users are coherent in the choice of the username by computing the edit distance and the complement of the Jaccard Index on the pairs $({u_{i}^{j}},{u_{i}^{k}})$ build from the set *U*_*i*_ of the usernames associated to the individual *i*. The former quantifies how dissimilar two strings are by computing the minimum number of operations to change ${u_{i}^{j}}$ into ${u_{i}^{k}}$. The allowed operation are the insertion, the deletion and the substitution of a character with another. The latter computes the Jaccard coefficient on the sets of characters of ${u_{i}^{j}}$ into ${u_{i}^{k}}$. In Figs. 21 and 22 we report the distribution of the above measures computed on each possible pair and in the inner figures the same quantities computed on randomly shuffled pairs. In both cases we obtain a peak at 0, indicating exact matches or the usage of the same character set, while the randomization shift the peak of the distributions towards values typical of dissimilar strings. However a portion of the pairs indicates that people may change the username across social sites. An example is shown in Fig. 23 where the change of the username between Pinterest and Google+ is more evident w.r.t. the average behavior reported in the previous figures. In fact we observe that the decision of changing the username depends on the pair of social sites.

## Concluding remarks

To face the new challenge of giving an all-around picture of people’s online behavior, in this paper we perform an analysis on the same users across multiple social media. Our study relies on a new rich dataset gathered from the social media aggregator Alternion. It collects information about how users post their favorite contents, their centrality on different social media and the usernames they choose. The study of this dataset let emerge two main insights. On the one hand we confirmed that the on-going phenomenon of the adoption of multiple social site is spreading. Not just people sign up in many social media but they are active and exploit their services as well. On the other, the temporal information about how and when users post allowed us to investigate how people manage the opportunity of having different communication channels at their disposal. As far as we know this study represents the first attempt to deal with the people’s activities on multiple social media by using a large set of social platforms and users.

As regards the multiple adoption of social sites, the analysis of social media usage shows that Alternion data capture the typical trend in today’s users, despite the limitations discussed on the dataset section. The usage of multiple platforms is gaining momentum. In fact, half of the users signed up in at least three different social sites. This result stresses the importance of a multiplex approach when conducting studies which rely on online social networks. In fact people are expressing their identity and their behaviors through multiple communication media. This observation is further strengthened by the results about active users. The fact that 73 % of active users publish on at least two social sites means that people choose the right online channel to communicate and convey their contents. Also in this case the multiplex approach is fundamental in the extraction of people’s interests and preferences. To this aim we plan to exploit these first results to study how users build their social identities across their social platforms Kooti et al. (2012). In particular we will verify whether different social norms characterize the major social platforms and if users adapt their self-presentation to these norms, as the results about the choice of the username may suggest. Finally we will ask whether the identity of a single user expressed through the contents s/he publishes is persistent and coherent with the profile information.

The multiplex nature of the Alternion dataset allowed us to investigate the maintenance of users’ popularity and centrality across social sites. In fact, despite the plethora of communication media, individuals are not confident with each media in the same way. For instance, someone better expresses and communicates through video, others through texts or blogs. That may result into different levels of engagement with fans or friends and, consequently, in different degree centralities. To this we asked whether a person’s popularity is uniform across the social sites. The analysis led to not straightforward results and indicated that user’s popularity in a given social site barely corresponds or does not correspond at all to his/her popularity on another social platform. Nverethenless, a more evident positive correlation between the posting activities across social platforms exists. In the future we plan to analyze the reasons behind the weak correlations we observe. In particular we will verify whether the social norms of the online platforms and the services they provide impact on the centrality of the users.

The multidimensional and longitudinal nature of the dataset is fundamental in the understanding of the human dynamics in communication and online social networks since it offers the opportunity to study how people handle different media. The primary goal of our analysis was to characterize the time-series associated to the posting activity of the single users across multiple social platforms and unveil their statistical properties focusing on measures which describe their level of burstiness. The analysis of the burstiness moved in two directions: i) we classified the aggregated behavior of each single user; and ii) within each user we characterized the bursty behavior in each single social media. The results show that i) singularly each event sequence keeps an high level of burstiness, i.e. the bursty behavior of the aggregated interaction sequence is the union of bursty event sequences and; ii) a period of high activity in the aggregated sequence does not imply that the user is highly productive in each social media during the same period. These observations represent the basis for a model of temporal patterns in multilayered social network which combines the idea of attention allocation and novel models which reproduce the human bursty dynamics Perra et al. (2012). We also plan to investigate the interaction sequences through frequent pattern analysis in order to highlight whether users are characterized by specific usage subsequences, i.e. they have a predefined scheduling in the usage of their preferred social media. The above analysis will be combined to the content analysis of the posts to see if personal topics change along the observation period or users change their posting behavior in terms of covered subjects or whether some social platforms specialize on specific subjects.

## Endnotes

^1^ http://www.pewinternet.org/2015/01/09/social-media-update-2014/

^2^ http://www.alternion.com

^3^ http://www.alternion.com

^4^ http://census.gov

^5^ The dataset is available by e-mail.

^6^ Alternion account is included by default, so counting starts from 1

^7^ *τ*_*b*_ takes into account ties.

^8^ *δ* is the Heaviside function
